# Phyto-Immunotherapy, a Complementary Therapeutic Option to Decrease Metastasis and Attack Breast Cancer Stem Cells

**DOI:** 10.3389/fonc.2020.01334

**Published:** 2020-08-07

**Authors:** Susana Fiorentino, Claudia Urueña, Paola Lasso, Karol Prieto, Alfonso Barreto

**Affiliations:** Grupo de Inmunobiología y Biología Celular, Pontificia Universidad Javeriana, Bogotá, Colombia

**Keywords:** breast cancer stem cells, natural products, immune response, polyphenols, phytomedicine, phyto-immunotherapy, tumor metabolism

## Abstract

In this review, we report on the complexity of breast cancer stem cells as key cells in the emergence of a chemoresistant tumor phenotype, and as a result, the appearance of distant metastasis in breast cancer patients. The search for mechanisms that increase sensitivity to chemotherapy and also allow activation of the tumor-specific immune response is of high priority. As we observed throughout this review, natural products isolated or in standardized extracts, such as P2Et or others, could act synergistically, increasing tumor sensitivity to chemotherapy, recovering the tumor microenvironment, and participating in the induction of a specific immune response. This, in turn, would lead to the destruction of cancer stem cells and the decrease in metastasis.

**Source of Data:** Relevant studies were found using the following keywords or medical subject headings (MeSH) in PubMed, and Google Scholar: “immune response” and “polyphenols” and “natural products” and “BCSC” and “therapy” and “metabolism” and “immunogenic cell death.” The focus was primarily on the most recent scientific publication.

## Introduction

The antitumor activity of natural products derived from plants is estimated to occur through the combination of various phytochemicals acting synergistically, rather than by isolated molecules, which alone may have greater toxicity and not exert the same activity. Additionally, these mixtures have specificity against their molecular targets, which, although difficult to identify, are considered drugs in themselves. These molecular targets may be found in events related to the prevention of carcinogenesis, the destruction of tumor cells directly, the modulation of the tumor microenvironment, the activation of the specific anti-tumor immune response, the induction of epigenetic changes, or the improvement of quality of life of the patient (1, 2). This plethora of activities is particularly relevant in the control of metastasis, where the breast cancer stem cells (BCSCs), are the main ones involved. In this review, we will focus on the role of these complex extracts on BCSC control through the modulation of energetic metabolisms and immune system activation.

## Breast Cancer Overview

Breast cancer is the most frequent tumor in women worldwide, accounting for 2.1 million new cases estimated for 2018 and 626.679 associated deaths for the same year, according to Globocan (3). Approximately one in 4 of all new cancer cases diagnosed in women worldwide is breast cancer, being the most common in 154 of the 185 countries polled. Breast cancer is also the main cause of cancer death in women (15.0%), followed by lung cancer (13.8%) and colorectal cancer (9.5%), which are also the third and second most common type of cancer, respectively; cervical cancer ranks fourth in both incidence (6.6%) and mortality (7.5%) (3, 4).

Regarding the origin of tumor cells, there are two models: the clonal evolution model (in which mutations accumulate and epigenetic changes occur in tumor cells, increasing survival of the fittest and most adaptable cells), and the cancer stem cell model (in which only the precursor cancer cells initiate and sustain tumor progression). Cancer stem cells (CSC) can also evolve clonally, so the two models are not mutually exclusive (5). At the molecular level, there is evidence showing that breast cancer evolves along two divergent molecular pathways of progression, primarily related to estrogen receptor (ER) expression, tumor grade, and proliferation. In addition, the identification of breast cancer susceptibility genes has shed light on some aspects of the pathogenesis of sporadic and inherited breast cancer (6).

The presence of specific markers in breast cancer such as ER, progesterone receptor (PR) and epidermal growth factor receptor 2 (HER-2) is useful to define the type of treatment indicated and the prognosis of the disease (7). ER and PR were the first biological markers evaluated, representing 50–75% of ductal carcinoma *in situ* (DCIS) lesions. The expression of ER correlates with the degree of DCIS and invasive breast cancer (IBC), and makes this type of cancer more sensitive to endocrine treatment and chemotherapy. In contrast, ER negative tumors have a higher proliferation rate and therefore greater aggressiveness (8). HER-2 plays an important role in the activation of HER-dependent cell growth and is over-expressed in 20–25% of IBC and 10–25% of IBC ER+. Overexpression of this marker in IBC contributes to oncogenic transformation and is considered a marker of poor prognosis. On the other hand, triple-negative (TN) patients for these markers have a poor overall prognosis since they respond poorly to treatment, possibly due to the close relationship that tumor cells have with the microenvironment and the presence of a molecular phenotype similar to that of BCSCs, which favors relapse with distant metastases (9). Although chemotherapy is currently the main systemic treatment for these patients, they are highly resistant (10). Therefore, having therapies directed at CSCs will be essential to treat both metastasis and this highly TN aggressive tumor.

Cancer staging system from breast cancer is based on the guidelines of the American Joint Committee on Cancer (AJCC) (11). Different therapeutic strategies are currently used in breast cancer treatment. These therapeutic strategies include, but are not limited to, local interventions (surgery and/or radiotherapy) and systemic treatments (chemotherapy, hormonal therapy, or targeted therapies) as anthracycline or taxane chemotherapy. The therapeutic options are limited when the primary response to chemotherapy is low. Patients with tumor progression or resistance are treated with Capecitabine, Vinorelbine, Gemcitabine, or albumin-bound Paclitaxel, however, these modifications have a low response rate (20–30%) with an average duration of survival of fewer than 6 months. Breast cancer patients undergoing prolonged chemotherapy treatment frequently develop resistance to various structurally related compounds, known as cross-resistance or multi-drug resistance (12). There are countless biological reasons for chemotherapy failure, which are heightened by the intrinsic heterogeneity of breast cancer cells present in the tumor (13). The presence of CSCs, which can be discovered from the development of the primary tumor, but can also be selected by treatment, aggravates this panorama due to their greater resistance to chemotherapy and radiation, contributing to therapeutic failures. Some studies have shown that CSCs are responsible for tumor formation and progression due in part to its self-renewal characteristics (14) and the expression by themselves of some other key factors implied in metastatic progression in breast cancer (15–17).

The first CSCs of solid tumors were identified in breast cancer (18) and later they were isolated in other organs. Al-Hajj et al. were the first to describe this subpopulation with the potential to form tumors within immunodeficient non-obese diabetic mice (NOD) with severe combined immunodeficiency (SCID) (18). Different surface markers such as CD44^+^/CD24^low^/EpCAM^+^ and lineage markers (negative for CD3, CD2, CD10, CD16, CD18, CD31, CD64, and CD140b) CD133^+^, CD49f^+^ and ALDH1 (19, 20), have been used for the BCSC identification from primary isolated tumors or metastases (18, 21, 22). Human BCSCs are identified by the ability to form spheres in low-adherence cultures, called mammospheres (14). Both normal and BCSC express the enzyme aldehyde dehydrogenase (ALDH) (22), however the expression of this ALDH seems to differentiate BCSC with epithelial phenotypic characteristics (ALDH^+^) which are more proliferating, from BCSC with mesenchymal characteristics (ALDH^−^) that have a greater invasive capacity but less proliferation. Furthermore, these cells demonstrate high plasticity that allows the transition between these two stages, thus increasing their aggressiveness (23). ALDH1 has also been widely used to identify CSCs, as well as the overexpression of ATP-binding cassette (ABC) transporters (24–26), belonging to the multidrug resistance proteins (MDR), in various types of cancer, including leukemias (27), colon (28), lung and pancreas (29, 30), among others. In fact, these MDR proteins are responsible for the expulsion of the Hoechst 33342 dye in the so-called “*side population*” (SP), which is also a phenotypic characteristic of CSC (31). CSCs can also be characterized by their ability to form spheres in culture (mammosphere assay for breast cancer), which is useful in enriching the BCSC population *in vitro* for in-depth study. Ponti and colleagues were the first to spread CSC from three breast cancer lesions and from an established breast carcinoma cell line, which grew *in vitro* in non-adherent cultures (14). This method allows for studying the properties of BCSCs as well as designing therapeutic strategies that target this type of cell (30).

We have recently shown in our laboratory, in a cohort of 78 patients with breast cancer, that those classified as Luminal B and TN who received neoadjuvant therapy before surgery, present an increase in the frequency of BCSC with a CD44^+^/CD24^−^/EPCAM^+^/CD49f^+^ phenotype together with the expression of ALDH compared to healthy donor tissue. Additionally, the correlation between ALDH expression and the breast cancer resistance protein (BCRP) was only observed in TN patients (unpublished data). An increase in the CD44^+^/CD24^low^ population has been observed in patients who have undergone anthracycline/cyclophosphamide or taxane chemotherapy, regardless of molecular subtype (32). In another study, an increase in the proportion of ALDH-1 cells was demonstrated in a cohort of 52 patients who had previously received neoadjuvant chemotherapy and had no complete pathological response. However, this increase was not observed when using the CD44^+^/CD24^−^ markers via immunohistochemistry (33). These results show that although chemotherapy is the first therapeutic option, BCSC enrichment is a risk factor that favors the appearance of metastasis a posteriori, and in many cases, the death of patients (34).

In a recent study, we also showed that the standardized extracts from *Caesalpinia spinosa* (P2Et) and *Petiveria alliacea* (Anamu), both of which present antitumor activity *in vitro* and *in vivo*, previously reported by our group (35–41), decreases the viability of CSC-enriched human tumor cell mammospheres, induces immunogenic cell death (ICD), in a better way than doxorubicin (42), and slows tumor appearance *in vivo* in immunodeficient mice (unpublished data). Furthermore, in a murine model of breast cancer, P2Et acts synergistically with doxorubicin reducing tumor growth, possibly due to its ability to inhibit the function of the Pgp multidrug resistant pump (39). These results may be promising, even more so when several studies have shown that chemotherapy can increase the population of the stem phenotype, and that co-treatment with plant extracts might increase sensitivity to therapy.

## Metabolism Of Breast Cancer Stem Cells

Cells under normal conditions have a low replication rate and use mitochondrial metabolism as an energy source, generating ATP through the tricarboxylic acid cycle (TCA) and oxidative phosphorylation (OXPHOS). By contrast, tumor cells need to increase the biosynthesis of macromolecules due to their high replication rates, for which they increase glucose uptake and use glycolysis as the main energy source, producing an increase in lactate levels even in the presence of oxygen. This process of increasing aerobic glycolysis is known as the Warburg effect (43). Although CSCs have been reported to increase the expression of markers that promote proliferation, differentiation, and metastasis, it is unclear how their metabolism works, in part because of the senescent characteristics they present. Initially, in CSCs generated from breast cancer lines, it was observed that they presented a higher glucose uptake with an increase in glycolysis, which in turn increased the synthesis of macromolecules and reactive oxygen species (44). Additionally, glucose availability has been shown to induce expression of specific genes in CSCs associated with glycolytic metabolisms such as c-Myc and Glut-1 (45). Likewise, in colon cancer and glioma cells, it was found that CD44 regulates the phosphorylation of pyruvate kinase M2 (PKM2), a key enzyme in the glycolysis of tumor cells, and thereby increases glycolytic metabolism, promoting an increase in glucose uptake and glutathione production to decrease the generation of reactive oxygen species (ROS) in hypoxic cells (46). These findings have suggested that low oxygen stresses and glycolytic metabolism are essential for the maintenance of CSCs in their undifferentiated state. In fact, hypoxia, in addition to increasing glycolysis, has also been considered a fundamental factor for CSC self-renewal, since it increases the activity of factors such as Oct4, c-Myc, and Nanog, important in maintaining the stem phenotype (47).

Contrasting with these assertions, it has been described that in embryonic stem cells, hypoxia increases metabolic plasticity, changing its glycolytic metabolism to a more oxidative metabolism, characterized by an increase in mitochondrial mass and the production of ROS, which favors differentiation (43, 48). Additionally, the increase in OXPHOS has also been associated with the induction of pluripotency, suggesting that the state of metabolic plasticity is necessary for the maintenance of pluripotent capacity in stem cells (49). These changes can also occur in CSCs, for which the microenvironment seems to play a fundamental role both in the generation and maintenance of CSCs and in their metabolic plasticity. The generation of metabolites such as lactate and free fatty acids by highly proliferating cells serves as fuel for anabolic cells, which would give an advantage at times when low availability of nutrients occurs, such as during chemotherapy (49). It has also been described that CSCs resistant to therapy have an increase in mitochondrial mass, which suggests an increase OXPHOS metabolism (50). This is related to a recent work showing that 4T1 cells with higher metastatic potential are metabolically more active compared to less metastatic cells. Additionally, it was observed that the metastases generated by 4T1 cells had different metabolisms depending on where they were established. This suggests that metabolic plasticity is necessary for CSCs to easily adapt to the microenvironment in which they are found and to give rise to highly proliferating cells (51).

This evidence suggest that CSC population may require a double-hit treatment, initially inhibiting glycolysis, which would result in increased phosphorylative activity, for which specific therapy for mitochondria, such as mitocanes (52–54) would then be used. This sequential double molecular target treatment would allow more effective attack on CSCs, reducing their plasticity and therefore their survival (55). In this sense, we previously showed that a *Petiveria alliacea* extract induces apoptosis of the murine breast cancer 4T1 cells through caspase-3 activation, DNA fragmentation without mitochondria membrane depolarization, and decreases in cell colony growth capacity. Changes in glycolytic enzyme expression, including reduction of PKM2, lead to a decrease in glucose uptake and lactate production, related with tumor regression in BALB/c mice transplanted with GFP-tagged 4T1 cells (37). Later we were able to show that *P. alliacea* extract entails the reduction in β-F1-ATPase expression, glycolytic flux triggering diminished intracellular ATP levels, mitochondrial basal respiration and oxygen consumption. Consequently, a decline in cell proliferation was observed in conventional and 3D breast cancer cells culture. Treatment of BALB/c mice transplanted with the murine breast cancer TS/A tumor showed that *P. alliacea* extract decreases the primary tumor growth and increases survival (56). It is interesting that we also observed antitumor activity on this mice breast cancer model with the P2Et extract (40, 41), even though the mechanisms of action of the two extracts, as well as their molecular composition, are diametrically opposed. In the case of *P. alliacea* extract, some of the main compounds are dibenzyl sulfide, 4-ethyl petiveral and glutamyl-S-benzyl cysteine, lignoceric acid and myricitrine. Additionally, in all the cell models studied, the anamu extract has a strong intracellular pro-oxidant capacity that is more or less intense depending on the cell type. In contrast, P2Et, mainly composed of derivatives of gallic acid induces cell death through mitochondrial depolarization, reticulum stress via PERK and increase of intracellular Ca^++^ without a remarkable increase in ROS (57). Unlike *P. alliacea* extract, P2Et has an important antioxidant capacity (39), however, both induce the expression of immunogenic death markers, possibly using different pathways.

BCSCs have shown plasticity that allows them to transition between a proliferative state that has been called epithelial-like (E) with high ALDH expression, and a quiescent, invasive mesenchymal-like state (M) characterized by CD44^+^/CD24^−^ phenotype. The balance between these two stages is modulated by the microenvironment (23). The participation of metabolism or oxidative stress in this phenomenon is not well-understood, but it has recently been shown that the M and E stages of the BCSC are related to different metabolic pathways and show marked differences in terms of sensitivity to glycolysis inhibitors or of redox metabolism. Metabolic or oxidative stress generated by 2-Deoxy-D-glucose (2-DG), H_2_O_2_ or hypoxia promotes the transition from a ROS^lo^-M-BCSC state to a ROS^hi^-E-state. This transition is reversed by N-acetylcysteine and mediated by activation of the AMPK-HIF-1α axis. E-BCSCs show a robust antioxidant response mediated by NRF2, making them vulnerable to ROS-induced differentiation and cytotoxicity resulting from NRF2 suppression.

In this sense, the activity of 2DG on TN breast cancer (TNBC) cell lines, the parental Hs578T, and it's more aggressive variant Hs578Ts (i) 8 was recently evaluated. Treatment with this glycolysis inhibitor showed inhibition of migration, invasion, and decreased ability to resist the anoikis of the more aggressive subtype Hs578Ts (i) 8, above the parental line. The aggressive line has a more glycolytic phenotype due to mitochondrial dysfunction and also has a higher proportion of cells with a CSC phenotype (58). Given the above, inhibition of glycolysis in conjunction with other metabolic modulators could be a useful therapy in the elimination of BCSC (59). Finding specific compounds targeting tumor metabolism has not been easy, but standardized complex extracts might be part of the solution, as we have been seeing it in our laboratory.

Other targets, such as phosphoglycerate dehydrogenase (PHGDH), are required for redox homeostasis, maintenance of BCSC, and lung metastases (60). It also participates in the resistance to sorafenib in advanced cellular hepatocarcinoma (61). This enzyme is a target of Ixocarpalactone A, obtained from diet tomatillo (*Physalis ixocarpa*) (62). Other compounds derived from natural products, such as turmeric, have been extensively studied, and their activity on the inhibition of different signaling pathways characteristic of BCSCs has been observed, as well as their ability to reverse multi-resistance to drugs through the inhibition of drug resistance pumps, decrease in the number of mammals *in vitro*, and increased awareness of chemotherapy treatment, among others (63).

Indeed, natural products such as phenolic compounds, isoprenoids and alkaloids target cellular metabolism (64). They have also been implicated in the inhibition of glycolysis, glucose transporters, and glycolytic enzymes such as GLUT1-4, Hexokinasa1-2, Pyruvate kinase M2, and lactate dehydrogenase (65). A study in mice showed that the content of ROS in stem cells of epithelial origin CD24^med^/CD49f^high^/Lin^−^ with high capacity for renewal is lower than its CD24^high^/CD49f^low^/Lin^−^ counterpart. In this same work, it was shown that the expression of genes coding for antioxidant enzymes in human BSCS obtained from primary tumors is higher than in their non-tumor counterpart, which was related to a lower content of ROS and a higher radioresistance (66). Given the complexity of these cells and the important influence of the tumor microenvironment in the generation and maintenance of the resistant and highly tumorigenic phenotype of CSCs, in general, therapy could be aimed at recovering the normality of the tumor microenvironment, which could allow CSCs to recover its normal phenotype as well as the sensitivity to chemotherapy and to the immune response (67, 68). Likewise, it is important to consider a mixed therapy that contemplates both the inhibition of glycolysis and the reduction of phosphorylation metabolism, in order to cover the tumor in all its heterogeneity.

Another important factor associated with the tumor microenvironment is the role tumor stromal cells play, for example as cancer-associated fibroblasts (CAF), which may be derived from tissue-resident fibroblasts or from mesenchymal cells recruited during growth-induced chronic inflammation tumors, in the maintenance of CSCs. CAFs, in addition to providing metabolism-derived by-products that support the growth of tumor cells (69), produce a good amount of cytokines that help promote the phenotype “stemness” in CSCs. For example, they secrete IL-6 that helps generate the mesenchymal epithelial transition phenotype involved with CSCs, or IL-8 that primarily regulates epithelial-like phenotype (E) with high ALDH expression (70). Therefore, CAFs become an alternative target for natural products due to their innate anti-oxidant activity, which could reverse the protumoral phenotype of these cells, leading them to reconstruct a normal microenvironment.

## Natural Products In The Control Of BCSC And The Induction Of Immune Response

Natural products can have antitumor activity through the elimination of the tumor cells themselves or indirectly through the activation of the antitumor immune response, as we will see later. Although its role on the BCSC is beginning to be understood, there is evidence about their role in controlling metastases or improving survival in patients with breast cancer (71), which means that they indirectly act by decreasing the tumorigenicity of BCSCs.

Plant extracts can act on multiple pathways that participate in the maintenance of BCSC (72), regulating tumor metabolism (64) or even by acting on the tumor microenvironment (73–75). There is a close relationship between the tumor microenvironment and tumor metabolism. This is reflected in the most important genetic alterations of CSCs, which are related to changes in tumor metabolism, such as by OCT4, KLF4, SOX2, and MYC. Meanwhile, NOTCH, WNT/β-catenin, PI3K/Akt, PTEN, NF-κB, KRAS, HIF, TP53, and other oncogenic pathways are related to the maintenance of the stemness capacity of CSCs, which could be a consequence of the first wave of genetic alterations (49).

PIK3CA mutations are found in patients with positive lymph nodes and subsequently manifest the mutation in the BCSCs present in the residual disease. Neoadjuvant therapy does not decrease cells with PIK3CA mutations, which appear to be more resistant to chemotherapy (76). In fact, tumors in which the BCSCs present defects in the signaling pathway PI3K/Akt are more predisposed to present nodal metastases. This marker is of utmost importance, and there are currently clinical studies evaluating the activity of some inhibitors of this pathway (77). Recently, it was reported that a group of compounds derived from pyrrolo pyrimidines have activity on PI3K and could act particularly on BCSCs (78). Another study found that fisetin, a dietary flavonoid, alone or in combination with 5-FU, affects tumorigenesis in the mammalian intestine. Treatment of cells with mutations in PIK3CA with fisetin and 5-FU decreases the expression of PI3K, the phosphorylation of AKT, mTOR, its target proteins, the constituents of the mTOR signaling complex and increases the phosphorylation of AMPKα, therefore possibly have a direct role on the BCSC (79).

On the other hand, hypoxia-inducible factor (HIF) is important in the selection and generation of BCSC, but also in the modulation of inflammatory response. In cancer, it has a primary role in PKM2 activation and aldolase A synthesis which in tumor cells play a fundamental role in the maintenance of glycolytic metabolism. HIF-1α is over expressed in several types of tumors, such as breast cancer, and conditional deletion of HIF-1α leads to a primary tumor decrease and metastasis, related with a reduction in BCSC frequency (80). HIF-1α can also regulate the interaction of BCSC with the microenvironment, placing it in a key position in maintenance of the tumor stem phenotype and suppression of immune response (81). Some natural compounds, such as diallyl trisulfides, reduce the expression of HIF-1α in breast cancer cell line (MDA-MB-231) inhibiting hypoxia-induced breast cancer metastasis (82). HIF inhibitors in clinical studies have shown modest results and high toxicity (83), therefore, HIF inhibition should be approached in a more holistic way, modulating for example the external factors that lead to HIF-1α activation in the tumor context.

Genetic signatures in BCSC responsible for self-renewal, such as c-KIT, TGF-β, the alpha 6 subunit of the integrin, STAT3, together with the Wnt/β-Catenin pathway which is also altered in TNBC (84, 85), are targets of Piperines (86). More generally, a recent study reviews how natural products can inhibit several signaling pathways involved in TNBC tumorigenesis or induce apoptosis through the inhibition of survival pathways activated by intrinsic tumorous cell disorders (87). This opens the door to continue studying how to use these natural products, preferably in combination with conventional chemotherapy that can modulate the signaling pathways that favor the maintenance of BCSCs.

In an *in vivo* model of murine breast cancer, called 4T1-H17, enriched with CSC-ALDH^+^, we showed that the anti-tumor immune response was the main element capable of controlling tumor progression and metastasis. Animals were vaccinated with 4T1-H17 cells previously treated with doxorubicin, a known ICD inducer (88), and fewer mice were found to develop primary tumors and macrometastasis, while inducing a multifunctional response of CD4^+^ and CD8^+^ T cells, suggesting that this treatment improved the control of highly metastatic and resistant 4T1-H17 tumor cells (89). A recent study showed that the cytotoxic T lymphocytes (CTL) response induced by autologous dendritic cells activated with antigens derived from BCSC significantly inhibits the proliferation of stem cells *in vitro* and decreases tumor size when treating transplanted mice with 4T1 breast cancer (90). Likewise, immunodominant epitopes derived from ALDH have been used to generate CD8^+^ T cells that specifically recognize and lyse human tumor cells of the breast, pancreas, head, and neck with elevated levels of ALDH1A1 (91, 92). The percentages of ALDH1A1 high cells decreased by 60–89% as a result of ALDH1A1-specific CD8^+^ T cells-mediated toxicity *in vitro*. In preclinical models using human tumor xenografts in immunodeficient mice, ALDH-specific CD8^+^ T cells inhibited the growth of xenografts and metastases, and prolonged survival after adopted (92). These studies show that CSCs are sensitive to T cell-mediated death.

P2Et has *in vitro* and *in vivo* activity against tumors generated by conventional 4T1 cells, but in the case of the 4T1-H17 line, despite having shown *in vitro* activity, both in 2D and 3D models, it did not show activity *in vivo* (89). The molecular mechanism by which P2Et acts is related to its ability to induce mitochondria-dependent apoptosis, with caspase activation, unfolded protein response (UPR) activated through PERK and cytoplasmic calcium increase, and its high intra and extracellular antioxidant capacity (57). The reason why P2Et does not present activity *in vivo*, despite inhibiting the formation of spheres *in vitro*, remains unknown. We can rule out the issue of bioavailability since it presents *in vivo* activity against 4T1 and B16-F10 cells (36, 40, 41), which is why the interaction of the tumor with the microenvironment must play a fundamental role. However, this question must be addressed in a tumor model in which the microenvironment is modified as the tumor develops, since it is this communication over time that generates this favorable space for the tumor growth and it is there where we can see if the P2Et extract (as well as other plant extracts) really plays a synergistic role in tumor therapy. This will also allow us to evaluate if this extract really favors the generation of an adaptive immune response.

The role of the microenvironment in the response of tumors to treatment with cellular stress modulators or antioxidants is evidenced in the works of Sayin, and Le Gal, where it is observed that antioxidants accelerate tumor progression and metastasis in a melanoma model and a transgenic model of lung cancer. This effect is related to an increase in glutathione synthesis induced by antioxidants such as N-acetyl cysteine (NAC) and Trolox (93, 94). In this regard, we recently showed that preventive treatment with P2Et of healthy BALB/c or C57BL/6 mice promotes tumor growth and death of these animals when 4T1 and B16-F10 tumors are subsequently transplanted, respectively. These facts are related to the generation of a pro-inflammatory environment in the treated animals, related to a preactivation of the immune response, which was clearly evidenced by a significant increase in plasma IL-6 (38) in contrast to previously observed experiments, where the P2Et decreased markers of poor prognosis such as IL-6 in the 4T1 model (41). A systematic review of the effect of antioxidants on the immune system showed that they significantly decreased tumor necrosis factor-alpha (TNF-α) production only in individuals who had a pro-inflammatory base condition, but no change in normal individuals (95). On the other hand, several natural products have been used as photosensitizer that when accumulated in tumors are activated by a light source during photodynamic therapy, increasing ROS and inducing cell death in tumor cells (96). Particularly, hyperacin, a natural product obtained from *Hypericum perforatum*, is directly accumulated in ER and after Dynamic Photo Therapy (PDT) it favors ROS production, ER stress response, calreticulin surface exposure and ICD (97). Typically, ROS production is associated with induction of ICD; however, it should be modulated as ROS produced in cancer cells can impact TME mediating immunosuppression via tolerogenic myeloid cells (98).

Microenvironment factors produced during tumorigenesis or even due to the inadequate activation of the immune response, favor the maintenance of CSCs. IL-6 induces the conversion of non-BCSC to BCSC by activation of OCT-4 transcription through STAT3. Likewise, STAT3 binds to the SOX2 and MYC promoter, increasing tumorigenicity, the efficiency of sphere formation, and ALDH activity. By decreasing HIF activation, reducing ROS due to the antioxidant activity of natural products, STAT3 activation is then decreased, minimizing the tumorigenic capacity of BCSCs. The decrease in IL-6 production could lead to a decrease in the conversion of non-BCSC to BCSC. Inhibition of downstream signaling by the IL-6 receptor has been shown to inhibit the growth of TNBCs, but not of its non-TNBC counterpart (99).

In previous studies, we observed that P2Et decreases the number of ALDH^+^ cells *in vitro* (39), although *in vivo* it presents a reduced activity to this ALDH^+^ population. However, we observed that treatment with P2Et delays the appearance of the tumor in a model of orthotopic transplantation of tumor cells of TNBC enriched in tumor cells with CSC phenotype (unpublished data). Our recent results are in line with a recent review showing that the antitumor effects of 10 Chinese plants and their bioactive compounds have immunostimulatory and cytotoxic activity against breast cancer cells, further reducing metastasis and improving the quality of life of patients. Among them are *Angelica sinensis* (AS, Dang Gui in Chinese), *Panax notoginseng* (PN, San Qi in Chinese), *Scutellaria barbata* and *Oldenlandia diffusa*, Licorice (Gan Cao in Chinese) and *Radix Salvia miltiorrhiza* (SM, Dan Shen in Chinese) (100). All this suggests the presence of a complex network of cellular interactions, which should be analyzed taking into account multiple variables that so far do not seem to have been taken into account.

An example of this complexity is the fact that MAPKs, essential in inflammation and cancer control, regulate cellular activities involved in tumor progressions such as proliferation, apoptosis, and escape of the immune response. Inhibitors of this route have been associated with adverse effects; however, in the context of prevention and treatment, it has been suggested that some dietary factors, such as virgin olive oil or nutraceuticals containing them, may interact with this route, being adjuvants of antitumor therapy (101).

Medicinal fungi such as *Ganoderma lucidum*, which has been widely studied and is called the immortality fungus, are also among the activators of the immune system. Its main components are the polysaccharides obtained from the aqueous extract, as well as the triterpenes obtained from the extract with organic solvents, which together seem to have direct antitumor activity and even more importantly, an activating activity of the immune response. The vast majority of studies found in the literature evaluate antitumor activity *in vitro* on tumor cells, showing some of the mechanisms involved, which may give evidence of their direct activity. However, few studies measure the activation of the immune response to the tumor, which can only be evaluated in *in vivo* experiments, in animal models with a complete immune system, or controlled clinical studies in cancer patients (102).

Regarding the activity of *G. lucidum* on BCSC, it was found that it decreases the viability of human tumor cells of TN phenotype, it reduces the gene and protein expression mainly of STAT3, as well as the phosphorylation of the protein, it slightly reduces the activity of ALDH1, and it decreases the formation of mammospheres. It has also been observed that some isolated Ganoderma compounds can inhibit the *in vitro* growth of quiescent cells with a low proliferation rate, at high concentrations, and *in vitro* (103).

When looking for clinical studies in NCBI with the keyword Ganoderma and cancer, we found only 3 studies, of which one of them was on head and neck cancer with no results (NCT02238587), another in prostate cancer that is ongoing (NCT03589781) and another in cancer in children (NCT00575926). It is possible that, not only for Ganoderma but for other natural products, the low availability *in vivo*, together with the lack of knowledge of the dose required to induce death, which is specific for each type of tumor, may explain why many of the clinical studies carried out so far have not given satisfactory results.

Although there is extensive literature on the role of natural products in the anti-tumor response, few studies have been carried out where the activity of plants in inducing tumor death is related to the activation of an appropriate anti-tumor immune response. The mechanism involved would be the induction of ICD in a tumor setting where the patient still has a functional immune response. Recently, we and others have shown that natural products can be the missing link by attacking tumor cells directly, improving the tumor microenvironment, and inducing activation of the immune response by mechanisms different from those previously reported for some chemotherapeutics that have the same activity (57).

**Figure 1:**
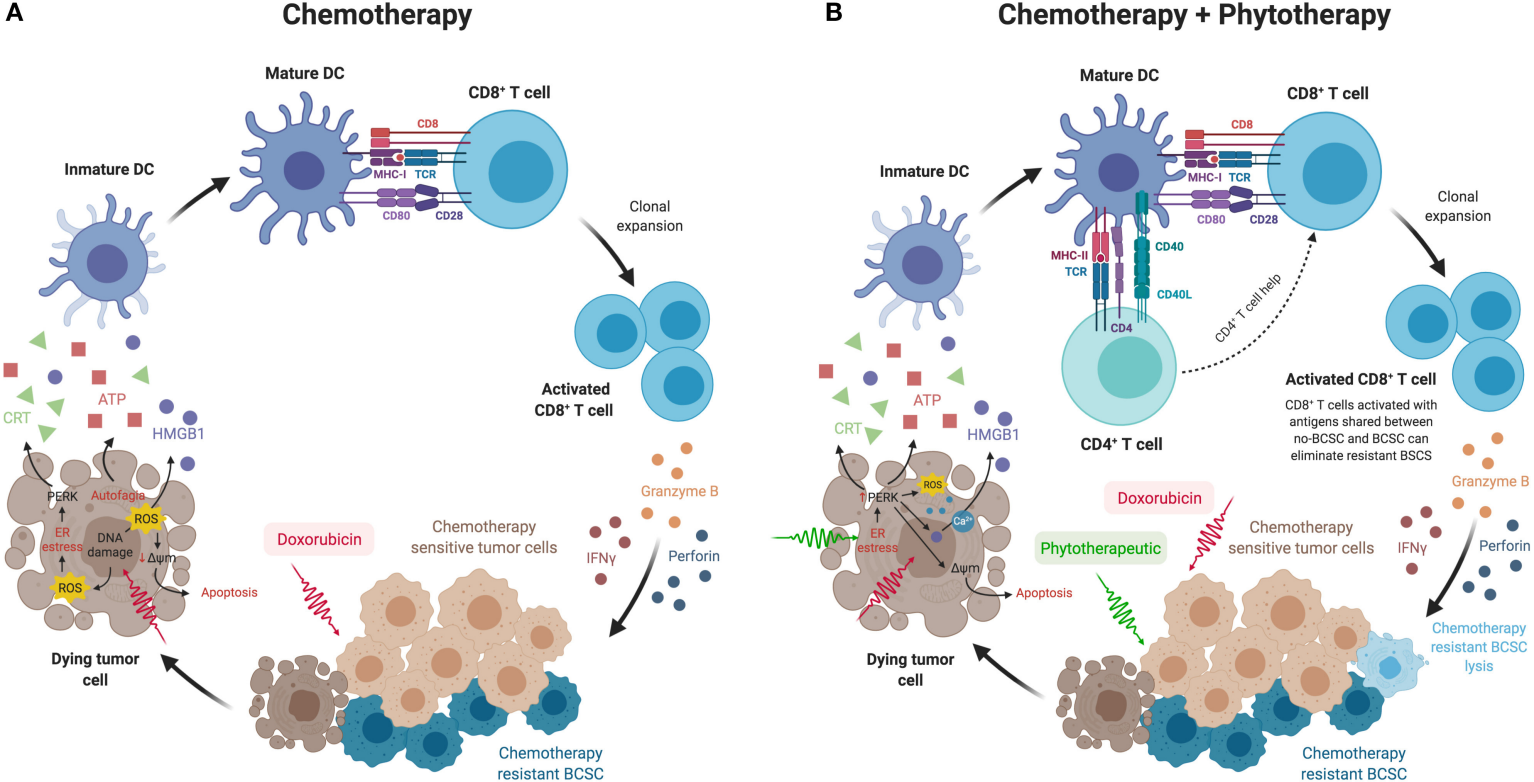
**(A)** BCSCs could be eliminated through the immune response generated *in situ* when chemotherapy-sensitive tumor cells die from mechanisms such as immunogenic cell death. This mechanism allows the activation of dendritic cells as a consequence of the release of danger signals from dead cells, such as calreticulin exposure on their surface, secretion of ATP, and HMGB1 release. The processing and presentation of tumor antigens released by the dead cells would allow the activation of CD8^+^ T cells by cross-priming mechanisms, which would ultimately attack the BCSC resistant to chemotherapy. **(B)** The combination of chemotherapy with phytotherapeutics or standardized extracts, such as P2Et, could act synergistically increasing tumor sensitivity to chemotherapy, recovering the tumor microenvironment and participating in the induction of an immune response not only of CD8^+^ T cells but also of CD4^+^ T cells, which in turn would lead to a better response of cytotoxic T cells that would attack BCSCs by cross-priming mechanisms, achieving lysis of BCSC and thus reducing metastasis. This figure was created using BioRender (https://biorender.com/).

Taking all the above, we could then think that BCSCs could be eliminated through the immune response generated *in situ* when chemotherapy-sensitive primary tumor cells die by mechanisms such as ICD. This mechanism allows the activation of dendritic cells as a consequence of the release of danger signals from dead cells. The processing and subsequent presentation of the tumor antigens released by the dead cells would allow the activation of CD8^+^ T cells lymphocytes by cross-priming mechanisms (104), which would ultimately attack the BCSC resistance to chemotherapy (Figure 1A). This can occur on the condition that CD8^+^ T cells recognize shared tumor antigens between chemotherapy-sensitive tumor cells and BCSCs, and also that BCSCs present these antigens in the context of MHC class I. This concept of endogenous or *in situ* vaccination should be explored in greater depth as a strategy to decrease metastasis and improve patient survival. The generation of this immune response *in situ* must also contemplate the reduction in immunosuppressive factors, that come from both the tumor cells themselves and the tumor microenvironment, that are released in the tumor microenvironment, and can be transformed by the presence of the tumor. The decrease in the deleterious inflammation, the ROS produced by cellular stress or even the regulation of tumor metabolism, could act as co-adjuvants, facilitating the effector action of the immune response. As presented in this review, natural products isolated or in standardized extracts, such as P2Et or others, could act synergistically, increasing tumor sensitivity to chemotherapy, recovering the tumor microenvironment, and participating in the induction of an immune specific response, which in the end would lead to the destruction of CSCs and the decrease in metastasis (Figure 1B).

## Evidence Of Immune Response Against Cancer Stem Cells

Although not all antigens that are over-expressed in CSCs are targets of immunotherapy, ALDH1A1 (92), survivin (105, 106), livina (107), and Bcl-2 (108), among others, have been reported to induce a specific immune response against CSCs. CSCs have been reported to renew after activation by Notch and amplification by Hedgehog and Notch signals of β-catenin (Wnt) (109). When CSCs renew or become inactive, Notch and Numb are degraded to peptides by the proteasome and are presented by HLA I molecules from tumor cells or T cells (110). Thus, the first report demonstrating that CTL can recognize and eliminate CSC populations was using CSC-enriched MCF-7 breast cancer and SK-OV-3 ovarian cancer cells (CD44^+^/CD24^lo^/CD133^+^) after treatment with 5-fluorouracil and paclitaxel. These cells were incubated with peripheral blood mononuclear cells previously activated with natural immunogenic peptides of the protein Notch-1 (2, 111) and Numb-1 (41, 86–93), finding a specific decrease in the CSC population in both models (110). Over-expressed enzymes that participate in metabolic pathways are considered a good antigenic target for the development of immunotherapies. For example, as previously shown, the enzyme ALDH1A1 has been shown to be an attractive target for the induction of adaptive immune responses against cancer (91, 92).

On the other hand, to consider the possibility of generating long-term protection, the immune compartment of T cells must be evaluated. An efficient way of inducing the generation of effector T cells against tumors in patients is through so-called vaccination *in situ*, where, by means of the intratumoral administration of different types of immunomodulators, the response of T cells is specifically induced or amplified in each patient (112). One of the *in situ* vaccination strategies that have strongly attracted attention in recent years is the induction of ICD by antitumor drugs such as anthracyclines. In ICD, a cellular stress response is induced prior to death by apoptosis accompanied by the generation of various danger signals or damage-associated molecular patterns (DAMPs), which ultimately promote an appropriate effector response by T cells (88). It is proposed that induction of the anti-tumor immune response through increased immunovigilance mechanisms can prevent the re-emergence of tumors from therapy-resistant cells such as CSCs. In this immunomodulatory microenvironment and in the presence of appropriate stimulation, CD8^+^ T cells proliferate and differentiate into CTL. Activated CTLs acquire the ability to produce cytokines, such as interferon gamma (IFN-γ) and TNF-α, and cytotoxic molecules, such as perforin and granzymes (113). Helper CD4^+^ T cells also play an important role in the development of anti-tumor immunity by improving clonal expansion of CTLs at the tumor site, preventing activation-induced cell death, and promoting the generation and maintenance of memory CTL (114).

However, it has been described that the immuno-evasive and immunosuppressive properties of CSCs can be an obstacle to inducing an effector immune response that can eradicate them. BCSCs express low levels of MHC I molecules (115), suggesting that these cells may evade the response of CTL. Furthermore, CSCs have a high expression of PD-L1, which is why they can inhibit the cytotoxic functions of T cells and, therefore, present a lower susceptibility to death (111). Still, various studies have shown that CSCs could be immunogenic in certain settings. Currently, different clinical studies have been developed that use dendritic cells loaded with CSC or mRNA lysates to vaccinate patients with lung (116), pancreas (117), glioblastoma (118), and breast cancer (119) among others. The results showed that vaccination induces a measurable and specific anti-tumor immune response without strong side effects, suggesting that patients might benefit from anti-CSC vaccination. Treatment with standardized extracts from plants (phytomedicines), which have a large number of molecules with synergistic and sometimes antagonistic activities, which give them unique characteristics, could have multiple benefits. On the one hand, it could decrease the tumor mass by acting directly on the tumor, modulating the tumor microenvironment allowing its reversion to a normal metabolic stage, and as a consequence, favoring the activation of the anti-tumor immune response by a mechanism that could be considered as vaccination.

## Author Contributions

SF, CU, PL, KP, and AB reviewed the literature and wrote the manuscript. PL prepared figure. SF contributed to the planning of the content and the critical evaluation of the text. All authors approved the final version of the manuscript.

## Conflict of Interest

SF and CU are inventors of a granted patent related to P2Et. The remaining authors declare that the research was conducted in the absence of any commercial or financial relationships that could be construed as a potential conflict of interest.
